# *In situ* shaped skin grafting following marsupialization for sacrococcygeal pilonidal disease: a retrospective case series

**DOI:** 10.1097/MS9.0000000000005704

**Published:** 2026-09-18

**Authors:** Yongchang Zhao, Yuan Tian, Xin Jin, Xiaoshuo Wu, Xun Shen, Feng Sun, Yuying Li

**Affiliations:** a Department of Colorectal Surgery, The First Affiliated Hospital of Guangzhou University of Chinese Medicine, Guangzhou, China; b Department of Colorectal Surgery, Weinan Central Hospital, Weinan, Shaanxi, China

**Keywords:** *in situ*-shaped skin grafting, sacrococcygeal pilonidal disease, surgical outcomes, unroofing and marsupialization

## Abstract

**Background:**

Optimal surgical management of pilonidal disease remains controversial, with existing techniques requiring trade-offs between recurrence risk and recovery time. We report our experience with unroofing and marsupialization combined with *in situ*-shaped skin grafting (SSG), focusing on perioperative outcomes, recovery, and recurrence.

**Methods:**

This case series included 53 consecutive patients who underwent SSG. Outcomes analyzed included operative parameters, recovery, recurrence, complications, and patient satisfaction.

**Results:**

The median operative time was 61 minutes (IQR, 45–70). The median hospital stay was 4 days (IQR, 3–6), with a median cost of 11 037.9 CNY (IQR, 10 519.0–11 944.0). Patients resumed pain-free activities after a median of 2 days (IQR, 2–4) and returned to work or school after 14 days (IQR, 10–15). Wound healing time was 22 days (IQR, 20–25). At the final follow-up, recurrence occurred in two patients (3.8%). No postoperative complications were observed. Sensory disturbance was reported in four patients (7.5%), none of whom reported interference with daily activities. Overall patient satisfaction was high, with a median score of 5 (IQR, 4–5).

**Conclusions:**

This single-center series demonstrates that unroofing and marsupialization, combined with *in situ* SSG, is a feasible surgical approach, offering rapid functional recovery and low recurrence rates without major complications. SSG may represent an intermediate option between prolonged healing by secondary intention and extensive flap reconstruction. Prospective multicenter comparative studies are warranted to establish optimal patient selection criteria and define the long-term role of this technique.

## Introduction

Sacrococcygeal pilonidal disease is a chronic inflammatory condition^[^1^]^ that predominantly affects young adult males and compromises quality of life. Western countries report an annual incidence of up to 26 per 100 000^[^2^]^, with German data demonstrating an increase from 29 to 48 per 100 000 between 2000 and 2012^[^3^]^. Reported incidence appears lower in Asian populations, although epidemiological data from China remain limited^[^4^]^. Disease recurrence has a substantial impact on long-term patient satisfaction^[^5^]^ and varies widely (0–100%) depending on surgical technique, patient selection, and duration of follow-up^[^6–8^]^, reflecting inherent trade-offs between recurrence risk, healing time, and procedural morbidity.

HIGHLIGHTS

- Shaped skin grafting combines marsupialization with *in situ*-shaped skin grafting.
- It was feasible in 53 consecutive patients with pilonidal disease.
- Median time to return to activities was 2 days.
- Wound healing was achieved in a median of 22 days.
- The recurrence rate was 3.8% at a 47-month follow-up.

Current surgical approaches face a fundamental dilemma: techniques minimizing recurrence often prolong recovery, whereas rapid-healing methods risk higher failure rates. Midline closure offers quick healing but recurs frequently (4–45%) with substantial wound complications (14–74%)^[^9–12^]^. Off-midline flap techniques (Karydakis, Limberg, cleft lift) achieve lower recurrence (0–23%)^[^9^]^ but require extensive tissue mobilization, are technically demanding, and involve prolonged immobilization^[^3,13,14^]^. Excision with healing by secondary intention minimizes recurrence but extends healing time^[^15^]^, causing prolonged functional limitation and reduced patient satisfaction^[^16^]^.

We developed an approach combining marsupialization with *in situ*-shaped skin grafting (SSG) to bridge this gap. The technique unroofs infected sinus tissue; marsupialization is achieved by suturing the wound edges to the sinus walls; and immediate coverage is provided using shaped grafts harvested from perilesional skin. This approach preserves healthy adjacent tissue while restoring the natal cleft contour. We conducted a single-center retrospective case series at the Department of Colorectal Surgery, assessing perioperative outcomes, recovery, recurrence, and patient satisfaction following SSG. In accordance with the IDEAL framework^[^17,18^]^, this series was designed to explore the feasibility and safety of SSG and to inform future comparative studies.

## Methods

### Study design and timeline

We conducted a retrospective case series at a tertiary referral center. The study included consecutive patients who underwent marsupialization combined with in situ SSG for sacrococcygeal pilonidal disease between October 2019 and October 2024.

The retrospective data collection and analysis were initiated in October 2025. Data were extracted from electronic medical records, encompassing demographic details, operative information, clinical examinations, and follow-up outcomes. To enhance outcome ascertainment and minimize loss to follow-up, a structured active follow-up program was conducted from October to December 2025, supplementing routine clinical follow-up data accumulated prospectively since surgery.

This study received approval from the hospital’s Ethics Committee (K-2026-021), with a waiver of informed consent owing to the use of anonymized retrospective data. All procedures conformed to the Declaration of Helsinki and STROBE guidelines^[^19^]^. Data security was maintained through restricted access by authorized personnel.

### Inclusion criteria

- Underwent unroofing, marsupialization, and *in situ* SSG procedure.
- Clinical diagnosis of sacrococcygeal pilonidal sinus.
- Preoperative imaging confirmation of the sinus tract type and extent.
- Postoperative pathological verification excluded other diseases.

### Exclusion criteria

Patients were excluded if they had any of the following:

- Active acute pilonidal abscess (resolved infection is eligible after 4 weeks post-drainage).
- Known immunodeficiency or immunosuppressive therapy.
- Severe malnutrition (albumin <30 g/L).
- Psychiatric disorders affecting compliance.
- Sacrococcygeal malignancy or severe skin infection.
- Coagulation disorders (INR > 1.5 or platelet count < 50 000/μL).
- Severe cardiopulmonary insufficiency (ASA ≥ 4).

### Operative procedure

All patients were operated under combined spinal-epidural anesthesia. After anesthesia, patients were placed in the right lateral decubitus position with a cushion between the legs and the buttocks retracted laterally using wide adhesive tape. The operative field was shaved and disinfected with povidone-iodine solution. Prophylactic broad-spectrum antibiotics were routinely administered 30 minutes before surgery.

Preoperative imaging (ultrasonography or magnetic resonance imaging) was used to guide the surgical plan by mapping the lesion and ruling out acute infection. Following this, the sacrococcygeal pilonidal area and pits were identified intraoperatively. Only after confirming that the surgical findings were consistent with the preoperative imaging was the definitive procedure performed. Each pit was cannulated with a probe or curved clamp to delineate the extent of the sinus tracts. A split-thickness, thin- to intermediate-thickness skin graft (typically 0.25–0.35 mm) was harvested from redundant, healthy perilesional skin within the operative field before excision. No separate donor site was created, as the graft was obtained from tissue adjacent to the planned wound area. A calibrated hand-driven instrument (e.g., a roller dermatome) was typically used, although power-driven dermatomes were also suitable. The harvested graft was immediately preserved in sterile saline solution for subsequent use.

Under probe guidance, the sinus tracts were laid open (unroofed) by incising the skin along the outer margin, allowing complete exposure while preserving the sinus base. Infected and necrotic tissue with embedded hair was meticulously curetted, avoiding exposure of underlying adipose tissue. Precise hemostasis was achieved using monopolar electrocautery, with care taken to minimize thermal spread to surrounding tissues. The wound was then irrigated sequentially with povidone-iodine solution and sterile normal saline.

The wound was marsupialized by suturing the wound edges to the curetted fibrous pilonidal tract using continuous 4-0 Prolene sutures, thereby reducing the defect size and elevating the natal cleft. The preserved skin graft was meticulously tailored to match the dimensions of the wound bed, ensuring complete, tension-free coverage. Unlike traditional stamp grafting, SSG involves tailoring the graft specifically to the wound’s contour to optimize adherence and minimize dead space. The graft was then secured to the wound bed to ensure stable integration and prevent graft mobility. Graft viability was assessed at the first postoperative dressing change, typically performed between postoperative days 5 and 7. In cases requiring extensive grafting, multiple small fenestrations were created in the graft to facilitate drainage and minimize the risk of seroma or hematoma formation beneath the graft.

Finally, the wound was dressed with sterile petrolatum gauze, fluffed gauze, and a sterile gauze wrap and secured with a moderate-pressure bandage to promote graft adherence and hemostasis. The key surgical steps are illustrated in Figure 1.

**Figure 1.**
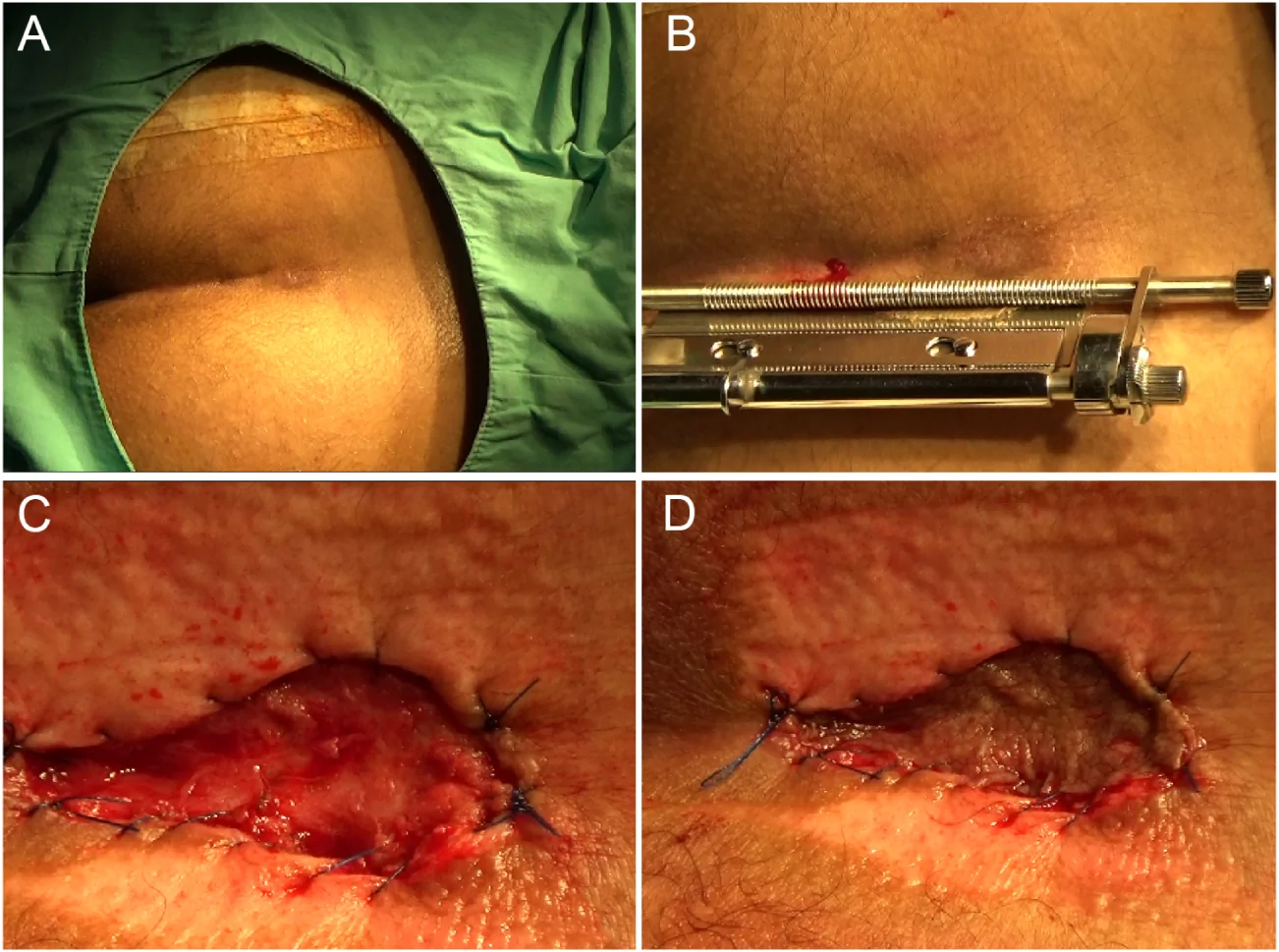
Surgical steps of unroofing and marsupialization combined with *in situ*-shaped skin grafting. (A) Preoperative appearance of sacrococcygeal pilonidal disease. (B) A medium-thickness skin graft was harvested for grafting. (C) Marsupialization elevates the natal cleft. (D) Immediate wound coverage with a tailored skin graft.

### Postoperative care

Patients received a single postoperative dose of broad-spectrum antibiotics. Additionally, they were prescribed celecoxib 200 mg orally twice daily for 7 days. Postoperative care included bed rest for 2 days to prevent graft displacement due to friction or pressure. Patients were typically considered for discharge between postoperative days 1 and 3. The initial dressing was removed between days 5 and 7 for graft viability assessment, and routine wound care was initiated. Subsequent outpatient management involved dressing changes every 1–2 days until complete healing. In cases where marsupialization with Prolene sutures was performed, the sutures were removed between postoperative days 10 and 12 (Fig. 2).

**Figure 2.**
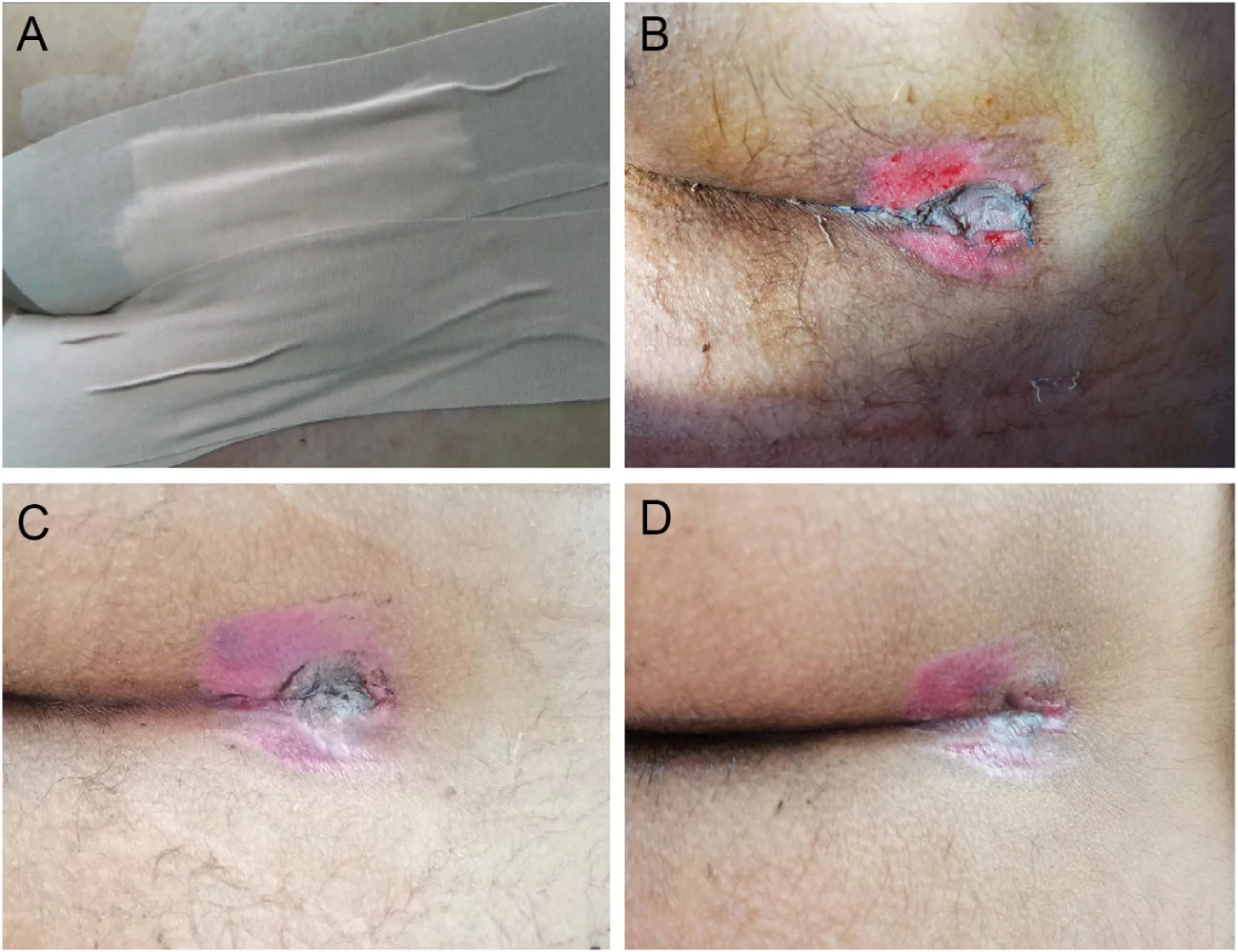
Postoperative wound healing and follow-up after SSG. (A) Immediate postoperative appearance. (B) Postoperative day 7. (C) Postoperative day 13. (D) Postoperative day 20.

### Outcomes

Recurrence was defined as active disease requiring intervention (medication, drainage, or excision) ≥30 days after SSG^[^12^]^, excluding early postoperative complications (e.g., infection or dehiscence within 30 days).

Secondary outcomes included operative time, length of hospital stay, costs, recovery indicators (time to return to normal activities, time to return to work, and healing time), complications, sensory disturbance, and patient satisfaction (assessed on a 1–5 scale).

Healing time was defined as the time to achieve complete epithelialization with no exudate or swelling.

Postoperative sensory disturbance was assessed based on patient self-report during follow-up and included symptoms such as numbness, hypoesthesia, or altered sensation in the surgical area. Sensory symptoms were further evaluated for their impact on daily activities.

Patient satisfaction was assessed using a numeric rating scale ranging from 1 (very dissatisfied) to 5 (very satisfied).

### Follow-up

Routine postoperative follow-up was conducted throughout 2019–2024. For retrospective analysis, a structured active follow-up (October–December 2025) supplemented existing data via telephone contact using standardized protocols and a comprehensive medical record review to identify complications or recurrences. Local patients underwent confirmatory physical examinations.

The median follow-up duration was 47 months (IQR, 33–57; range, 13–71). All patients completed at least 13 months of follow-up, a period considered adequate for detecting most early recurrences, although late recurrences cannot be excluded. The variable follow-up duration reflects the extended accrual period (October 2019–October 2024) and the timing of the retrospective analysis (October 2025).

### Statistical analysis

Continuous variables are presented as median (interquartile range [IQR]) and categorical variables as numbers (percentages). No inferential statistical testing was performed due to the descriptive nature of the study design. All analyses were conducted using SPSS (version 26.0, IBM, USA).

### Video and photographic documentation

Complete intraoperative videos were obtained, documenting the entire SSG technique from graft harvesting to final dressing. Serial postoperative photographs were taken during dressing changes and follow-up visits to record wound-healing progression. Representative videos and images are provided as Supplemental Digital Content material, available at https://links.lww.com/MS9/B389.

## Results

A total of 53 patients were included in this case series. The median age was 23 years (IQR, 20–26), and the majority of patients were male (84.9%). The median body mass index was 26.3 kg/m^2^ (IQR, 23.53–29.83), indicating that most patients were overweight. The median duration of disease was 6 months (IQR, 2–24).

Primary disease was present in 21 patients (39.6%), while 32 patients (60.4%) had recurrent disease. Most patients had one or two primary pits: 23 patients (43.4%) had one pit, and 18 patients (34.0%) had two pits. Three pits were observed in seven patients (13.2%), whereas no identifiable primary pits were present at the time of surgery in five patients (9.4%). Secondary openings were present in 49 patients (92.5%), with one opening in 47 patients (88.7%) and two openings in two patients (3.8%). Baseline characteristics of the study population are summarized in Table 1.

**Table 1:** Baseline characteristics of the study population (*n* = 53).

	SSG (*n* = 53)
Age (y)	23 (20–26)
Sex	
Male	45 (84.9)
Female	8 (15.1)
BMI (kg/m^2^)	26.3 (23.53–29.83)
Duration (months)	6 (2–24)
Disease status	
Primary	21 (39.6)
Recurrent	32 (60.4)
Primary pits (*n*)	
0	5 (9.4)
1	23 (43.4)
2	18 (34.0)
3	7 (13.2)
Secondary openings (*n*)	
0	4 (7.5)
1	47 (88.7)
2	2 (3.8)

Data are presented as median (interquartile range) or number (percentage), as appropriate.

BMI was calculated as weight in kilograms divided by height in meters squared.

Disease duration was calculated from symptom onset to surgery.

All patients underwent surgery without intraoperative complications. The median operative time was 61 minutes (IQR, 45–70); the median length of hospital stay was 4 days (IQR, 3–6); and the median total hospitalization cost was 11 037.9 CNY (IQR, 10 519.0–11 944.0). Postoperative recovery was rapid: patients resumed pain-free daily activities after a median of 2 days (IQR, 2–4) and returned to work or school after a median of 14 days (IQR, 10–15). The median wound-healing time was 22 days (IQR, 20–25).

During follow-up (median 47 months; range, 13–71 months), recurrence occurred in two patients (3.8%), both of whom were successfully treated with repeat SSG, and no further recurrence was observed. No major postoperative complications were recorded. As graft harvesting was performed from redundant perilesional skin within the operative field, no separate donor site was created, and no donor-site morbidity was observed. All patients demonstrated clinically viable graft incorporation at the first postoperative dressing change (postoperative days 5–7), with no evidence of graft failure. Four patients (7.5%) reported postoperative sensory disturbance, including numbness or altered sensation; however, none of these symptoms interfered with daily activities. Patient satisfaction was high, with a median satisfaction score of 5 (IQR, 4–5). Perioperative and postoperative outcomes are detailed in Table 2.

**Table 2:** Clinical outcomes of *in situ* shaped skin grafting.

	Value
Operative time (min)	61 (45–70)
Hospitalization costs (CNY)	11 037.9 (10 519.0–11 944.0)
Length of hospital stay (d)	4 (3–6)
Time to pain-free activities (d)	2 (2–4)
Time to return to work/school (d)	14 (10–15)
Healing time (d)	22 (20–25)
Follow-up duration (months)	47 (33–57)
Postoperative complications	
No	53 (100.0)
Yes	0 (0.0)
Recurrence at endpoint	
No	51 (96.2)
Yes	2 (3.8)
Numbness/sensation loss	
No	49 (92.5)
Yes	4 (7.5)
Numbness affecting daily life	
No	53(100.0)
Yes	0 (0.0)
Satisfaction score (*n*)	5 (4–5)
1	1
2	0
3	3
4	12
5	37

Values are presented as median (interquartile range) or number (%), as appropriate.

Recurrence was assessed at the last follow-up visit.

## Discussion

The optimal surgical management of sacrococcygeal pilonidal disease remains controversial, as existing techniques entail trade-offs between recurrence risk, healing time, and procedural morbidity^[^8,20^]^. In this series of 53 consecutive patients, unroofing and marsupialization combined with SSG was associated with a reasonable operative time, rapid recovery, low recurrence, and no major complications. Current evidence on pilonidal disease surgery has been shaped primarily by randomized trials comparing closure techniques, Cochrane systematic reviews, large meta-analyses, and the 2024 European Society of Coloproctology guidelines^[^8,16,21–23^]^. However, no previous study has evaluated marsupialization combined with *in situ* SSG as a distinct surgical approach, underscoring the rationale for the present exploratory investigation.

The observed recurrence rate (3.8%) appears comparable to that reported for established approaches. Primary midline closure achieves rapid initial healing but is associated with recurrence rates of 4–45% and wound complication rates of 14–74%^[^16,21,24^]^. A meta-analysis of 89 583 patients reported that long-term midline closure recurrence reached 67.9% at 240 months^[^8^]^, while a 2008 Cochrane Review confirmed significantly higher rates of surgical site infection (RR, 3.72) and recurrence (Peto OR, 4.54) compared with off-midline techniques^[^21^]^.

Off-midline flap techniques are associated with low recurrence rates, with reported rates of 0.2% at 12 months and 0.6% at 24 months for Karydakis and Bascom procedures, and 0.6% at 12 months and 1.8% at 24 months for Limberg flaps^[^8^]^. However, these flap-based procedures require extensive tissue mobilization and are technically demanding. Reported hospital stays average 3–4 days, with return to normal activities ranging from 8 to 28.6 days for Karydakis procedures and from 8 to 56 days for various off-midline techniques^[^22,25^]^. Bozkurt *et al* reported a mean hospital stay of 4.1 days following Limberg flap surgery and a mean time to return to work of 17.5 days^[^26^]^.

Excision with secondary-intention healing is associated with relatively low recurrence rates (2–42%)^[^16,21^]^ but is accompanied by prolonged healing times ranging from 21 to 92 days^[^16,21,27^]^. In a series of 570 patients, Burney reported median healing times of 46 days in women and 51 days in men^[^28^]^. Despite lower recurrence rates, the extended healing period may result in prolonged functional limitation and reduced patient satisfaction^[^5^]^.

SSG may represent an intermediate option between healing by secondary intention and off-midline flap procedures. In this series, the 2-day return to normal activities appears shorter than that reported for flap-based techniques, while the observed recurrence rate of 3.8% at a median follow-up of 47 months falls within the favorable range reported for off-midline approaches. In addition, the median healing time of 22 days compares favorably with secondary-intention healing (46–51 days), while avoiding the need for extensive tissue mobilization.

The pathogenesis of pilonidal disease is thought to involve loose hair acting as an “invader,” mechanical forces promoting hair insertion, and deep natal cleft anatomy^[^29,30^]^. Karydakis described these factors in his pathogenic formula, based on observations from more than 6000 patients^[^30^]^. Natal cleft depth has been identified as an important risk factor, with affected patients demonstrating deeper clefts compared with controls^[^31^]^.

Marsupialization converts deep, poorly drained sinus tracts into shallow, well-vascularized wound beds, thereby reducing wound depth and facilitating effective drainage^[^32,33^]^. Suturing the wound edges to the sinus base flattens the natal cleft, which may reduce hair accumulation, maceration, and mechanical irritation^[^30,34^]^. The addition of a shaped skin graft provides immediate epithelialization, establishing a continuous protective barrier during the early and vulnerable healing phase. In this series, clinically viable graft incorporation was observed in all patients, suggesting the technical feasibility of immediate graft coverage using SSG. In contrast to marsupialization alone, which typically requires 3–8 weeks of healing by secondary intention^[^32,35^]^, immediate graft coverage shortens the epithelialization period. Bascom demonstrated that midline pits represent enlarged hair follicles serving as portals for hair entry^[^29,36^]^; therefore, early epithelial coverage may help prevent follicular recolonization and reduce the risk of chronic inflammation.

From a biomechanical perspective, SSG may help redistribute shear forces away from the midline and reduce mechanical stress across the healing wound. Recent biomechanical studies have demonstrated that the lower sacrococcygeal region experiences substantial mechanical forces during sitting, with measured pressure reaching 106.6 mm Hg and shear stress up to 11.6 N, particularly in the horizontal direction^[^37^]^. These forces are amplified by the repetitive bending and sitting actions that tighten the gluteal skin and create negative pressure, driving hair deeper into the sinus^[^1^]^. Midline closures place wounds directly within this high-stress zone, predisposing to dehiscence and recurrence^[^8,16^]^. Off-midline techniques modify local anatomy by flattening the natal cleft to a clinically meaningful extent, a change consistently associated with lower recurrence rates across multiple surgical approaches^[^30,34,38^]^. The marsupialization component of SSG achieves similar cleft flattening by suturing wound edges to the sinus base, thereby reducing the depth and altering the mechanical environment. While the specific biomechanical properties of the grafted dermal component have not been directly measured, the provision of immediate epithelial coverage may theoretically reduce friction and minimize repetitive microtrauma during the vulnerable healing period, potentially mitigating stress concentration compared with healing by secondary intention or tensioned midline closures.

SSG appears to preserve native tissue planes and maintain local microvascular circulation, in contrast to flap-based techniques that require extensive undermining and may disrupt lymphatic drainage^[^39^]^. This relative tissue preservation may help explain the earlier return to normal activities observed in this series (median, 2 days), compared with reported recovery times of 8–56 days for flap approaches, as well as the absence of major complications.

The advantages of SSG may become more apparent in larger or more complex defects, in which healing by secondary intention is associated with prolonged epithelialization^[^5,28^]^. In this cohort, a substantial proportion of patients had recurrent disease (60.4%) and an elevated body mass index (median, 26.3 kg/m^2^), both of which are recognized risk factors for delayed healing and recurrence^[^2,8^]^. The acceptable outcomes observed in these recurrent or complex cases suggest that SSG may be a useful option for patients with previously treated or otherwise challenging disease.

Compared with off-midline flap procedures, which typically require 5–9 days of bed rest with restricted sitting^[^32,40^]^, patients undergoing SSG resumed normal activities after a median of 2 days. By preserving the natural surface anatomy and avoiding extensive reconstruction, SSG may reduce cosmetic concerns^[^32^]^ while facilitating faster functional recovery. In contrast, traditional marsupialization requires an average of 5.4 weeks before returning to work^[^41^]^, whereas patients treated with SSG returned to work after a median of 14 days, supporting the potential role of immediate graft coverage in accelerating postoperative recovery.

This study has several limitations. The single-center, retrospective design without a control group limits causal inference and generalizability. Variable follow-up duration and partial reliance on telephone follow-up may have underestimated late recurrences. The absence of validated patient-reported outcome measures precludes comprehensive quality-of-life assessment; future studies should incorporate validated instruments such as the Pilonidal Disease Activity Score or the Dermatology Life Quality Index to enable standardized cross-study comparisons. Potential learning curve effects across the extended accrual period, along with the lack of detailed anatomical measurements including cleft depth quantification, further constrain interpretation. These limitations highlight the preliminary nature of the findings and underscore the need for prospective comparative trials.

In conclusion, this case series suggests that SSG is a feasible surgical technique associated with rapid recovery, low recurrence, and no major complications. The proposed biological mechanisms – including wound environment modification through marsupialization, immediate epithelialization via grafting, cleft depth reduction, and tissue preservation – provide a plausible theoretical basis for these outcomes. Prospective, multicenter comparative studies with standardized outcome measures are warranted to define long-term efficacy and optimal patient selection.

## Data Availability

The datasets generated and/or analyzed during the current study are available from the corresponding author on reasonable request.

## Research registration unique identifying number (UIN)

Not applicable. This study is a retrospective case series and was not registered in a research registry.
